# Do parents of children with congenital malformations have a higher cancer risk? A nationwide study in Denmark

**DOI:** 10.1038/sj.bjc.6600488

**Published:** 2002-08-27

**Authors:** J L Zhu, O Basso, H Hasle, J F Winther, J H Olsen, J Olsen

**Affiliations:** The Danish Epidemiology Science Centre, University of Aarhus, Vennelyst Boulevard 6, 8000 Aarhus C, Denmark; Department of Pediatrics, Skejby Hospital, Aarhus University Hospitals, Denmark; Institute of Cancer Epidemiology, Danish Cancer Society, Copenhagen, Denmark

**Keywords:** cancer, congenital malformation, cleft lip and palate, case-control study, record linkage

## Abstract

To investigate whether parents of children with congenital malformations more often developed cancer after birth of the child, a population-based case-control study in Denmark was undertaken. By linking the Cancer Registry with the Central Population Registry, we identified 8783 cancer patients having their first child born between 1977 and 1995 before the cancer was diagnosed. Parents of 41 206 firstborn children of a 10% random sample of newborns from the Birth Registry between 1980 and 1995 were identified as controls. We obtained malformation diagnoses of children of cases and controls by linking to the Hospital Discharge Registry. We estimated the association between malformation and cancer by using logistic regression, adjusting for maternal age at birth and sex of child. We found no increased risk of cancer in parents having children with malformations in general, but a higher cancer risk in parents of children born with cleft lip/palate, odds ratio (OR) for all cancer=1.8 (95% confidence interval 1.0–3.2), OR for lymphomas=4.2 (1.3–13.5) and OR for leukaemia=8.1 (2.0–33.7). This association was not restricted to cancer cases diagnosed shortly after birth of the child. Our results suggest a common genetic association between these diseases, but further studies are needed.

*British Journal of Cancer* (2002) **87**, 524–528. doi:10.1038/sj.bjc.6600488 www.bjcancer.com

© 2002 Cancer Research UK

## 

We hypothesized that parental cancer and congenital malformations in offspring may correlate for the following reasons: (1) Some exposures may be teratogenic as well as carcinogenic and be present for parents as well as their children. Radiation may be such an exposure (Vorobtsova, 1989). Vinyl chloride (Infante *et al*, 1976) and oestrogen (Mclachlan *et al*, 2001; Titus-Ernstoff *et al*, 2001) have also been suggested to cause both congenital malformations and cancer. (2) The cancer process may be present in a preclinical state at the time of conception, and the disease could alter metabolism and distribution of important determinants for certain congenital malformations such as hormones or vitamins (Vainio, 1989).

In this paper we examined whether parents of children with congenital malformations have an excess of cancer after the birth of the child. If such association exists, this may help to focus further studies on causation of the cancers and malformations in question. We expected that only specific cancers and malformations would be associated, but we had no prior hypothesis for limiting the analyses to subsets of the diagnostic categories.

## MATERIALS AND METHODS

We used a case-control design. From the Cancer Registry, we first identified all male incident cancer patients who were born before 1983 (15 years old or more during 1977–1996) and female cancer patients born between 1926 and 1982 (15 years old or more but less than 50 years of age during 1977–1996). Patients who died before 1977 were not included. The Cancer Registry, established in 1943, includes all diagnoses made by clinicians or pathologists as part of the treatment or diagnosis. The register also includes cancer cases diagnosed post mortem (Storm *et al*, 1997). Cancer cases were classified according to the Danish Cancer Registry Code (The Danish National Board of Health, Statistical Office, 1999). The search yielded 380 341 patients with a cancer recorded for the first time from 1943 to 1996, identified by their personal identification number (CPR number), diagnosis of cancer (ICD7 codes) and time of diagnosis. The CPR number, which is unique to every resident, incorporates sex and date of birth and permits accurate linkage of information between registries.

Second, we identified all the cancer patients’ children born in Denmark up to 1995 by linkage with the Central Population Registry. The children were identified by the unique existing links between parents and children in the Central Population Registry. We had data on time of birth, birth order, place of birth and mothers’ citizenship. We then identified the first liveborn children who were born between 1977 and 1995 in Denmark to mothers with a Danish citizenship. A mother who has had cancer is more likely to be in the Cancer Registry if she has a Danish citizenship and her children are more likely to be born in Denmark. If the first liveborns were twins or triplets we included only the firstborn child. If the parents had a live born child before 1977, they were excluded from the study. We excluded 2534 children born after the cancer was diagnosed in their parents. We excluded 82 children where both parents were affected in the analyses. We also excluded parents with skin cancer (except melanoma), because it is known that skin cancers are underreported to the Cancer Registry. We ended up with 4938 first liveborn children to mothers with a subsequent cancer and 3845 first liveborn children to fathers with a subsequent cancer.

We identified as controls the parents of a 10% random sample of newborns from the Birth Registry who were born between 1980 and 1995 and whose mothers’ citizenship was Danish. We included only first live-borns and obtained 41 206 eligible pairs of parents. For these children we had similar data and from the same data sources as we had for cases.

We obtained the diagnoses of congenital malformations (ICD8 codes before 1994 and ICD10 codes from 1994), by linking the children of cases and controls to the Hospital Discharge Register by means of their CPR numbers. The Hospital Discharge Register started from 1977 as a population-based nation-wide registry with an average completeness over the study period of about 99% of all discharges from non-psychiatric hospitals (Andersen *et al*, 1999). Data on congenital malformations include all ICD8 codes 740 to 759 and ICD10 codes Q00 to Q99 as any discharge diagnoses made by midwives or any hospital physicians. We excluded the diagnoses of patent ductus arteriosus, undescended testis and hip dislocation due to the expected large variation in diagnosing these malformations. If a child was registered with more than one malformation, each malformation was counted. Cleft lip/palate are often classified into cleft lip with or without palate and cleft palate and they can also be an isolated malformation (nonsyndromic), or be part of a known syndrome (syndromic), e.g. trisomy 13 (Christensen, 1999). We searched our data for associated malformations in all cases of cleft lip/palate. Patients with major malformations or malformations known to be associated with cleft lip/palate were classified as syndromic cleft lip/palate.

From cases and controls we estimated cancer risk as a function of the prevalence of congenital malformation in offspring by means of logistic regression, taking maternal age at birth and sex of child into consideration. We made separate analyses; i.e. analyses including malformations at birth and analyses including malformations diagnosed within first year of life including those diagnosed at birth. We did subanalyses according to the parents’ gender, their type of cancer, age at time of cancer diagnosis and time from the birth of the child to the time of cancer diagnosis. We started by analysing all cancers and malformations grouped together. We then examined specific malformations and their relations to parental cancer. Finally, we subdivided the cancer diagnoses to further characterise results.

## RESULTS

For our identified 8783 parents who developed cancer subsequent to the birth of their first child, the five most frequent cancers were cancers of breast, melanoma, testis, cervix uteri, and brain and nervous system. The mean ages at diagnosis for males and females were 38.3 years and 36.0 years, respectively (range 17–85 years). The mean ages at birth of the child were 30.0 years for male cancer patients and 27.1 years for female cancer patients, 26.0 years for control mothers and 29.0 years for control fathers. The mean time interval from birth of child to diagnosis of cancer was 8.2 years for males and 8.9 years for females, range 0–19 years (Table 1

**Table 1:**
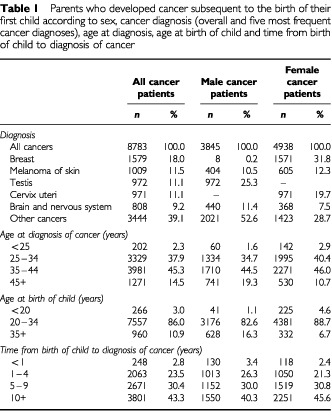
Parents who developed cancer subsequent to the birth of their first child according to sex, cancer diagnosis (overall and five most frequent cancer diagnoses), age at diagnosis, age at birth of child and time from birth of child to diagnosis of cancer

). The proportions of twins and triplets among first liveborns of male cancer patients, female cancer patients and the reference population were 1.1, 1.3 and 1.3%, respectively.

The prevalence of the restricted group of congenital malformations at birth in offspring of parents with subsequent cancer was 1.74%, compared to 1.86% in the reference population. Only cleft lip/palate was more frequent in offspring of parent who later developed cancer (Table 2

**Table 2:**
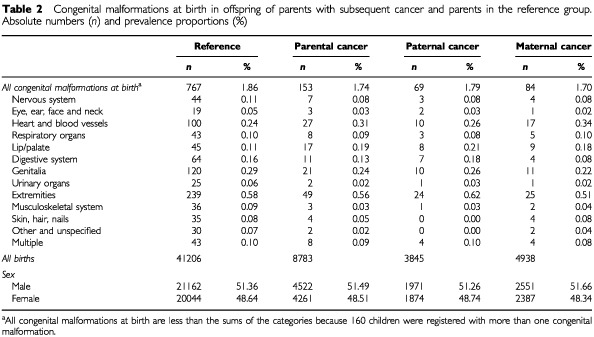
Congenital malformations at birth in offspring of parents with subsequent cancer and parents in the reference group. Absolute numbers (*n*) and prevalence proportions (%)

).

The odds ratios (ORs) for subsequent cancer in fathers, mothers or either parent of children born with cleft lip/palate were 1.9 (95% CI 0.9–4.1), 1.7 (95% CI 0.9–3.6) and 1.8 (95% CI 1.0–3.2), respectively, after adjustment for maternal age at birth and sex of the child (Table 3

**Table 3:**
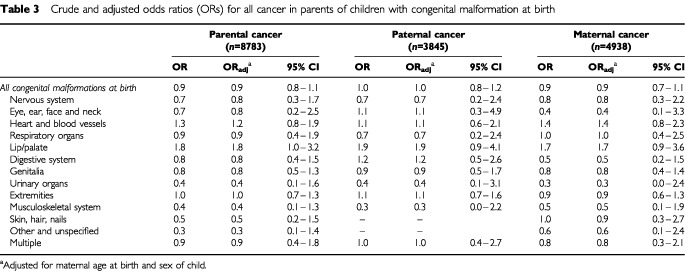
Crude and adjusted odds ratios (ORs) for all cancer in parents of children with congenital malformation at birth

). No other group of congenital malformations was significantly associated with subsequent parental cancers in either sex.

We checked whether cleft lip/palate in offspring was associated with an increased risk of any particular parental cancer (out of 10 major diagnostic groups). Parents of children with cleft lip/palate had a significantly higher risk of developing lymphomas or leukaemia. The ORs for parental lymphomas and leukaemia were 4.2 (95% CI 1.3–13.5) and 8.1 (95% CI 2.0–33.7), respectively, after adjustment for maternal age at birth and sex of child (Table 4

**Table 4:**
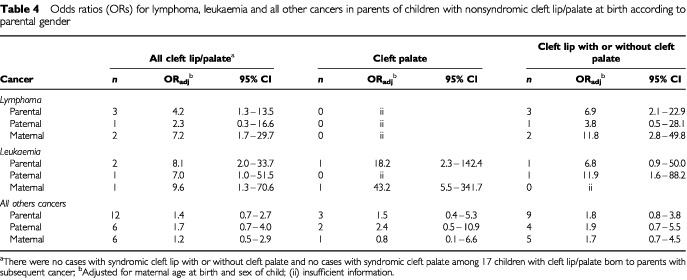
Odds ratios (ORs) for lymphoma, leukaemia and all other cancers in parents of children with nonsyndromic cleft lip/palate at birth according to parental gender

). Cleft lip/palate was not significantly associated with other parental cancers. Table 4 also shows that the OR for maternal lymphomas was associated with nonsyndromic cleft lip with or without cleft palate (OR=11.8; two observed cases) and the OR for maternal leukaemia was associated with nonsyndromic cleft palate (OR=43.2; one observed case), after adjustment for maternal age and sex of the child.

The association between cleft lip/palate and parental cancer was not restricted to cancer cases diagnosed shortly after the child was born with the malformation (Table 5

**Table 5:**
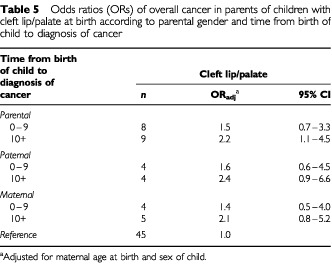
Odds ratios (ORs) of overall cancer in parents of children with cleft lip/palate at birth according to parental gender and time from birth of child to diagnosis of cancer

). The ORs remained unchanged if we included all malformations diagnosed during the first year of life (48% more malformations, data not shown).

Leukaemia was also more frequent in parents of children with congenital malformations in general. The ORs for leukaemia in father, mother, or either parent whose child was born with any recorded malformations were 2.1 (95% CI 0.9–5.1), 2.3 (95% CI 0.8–6.2) and 2.2 (95% CI 1.1–4.2), respectively, after adjustment for maternal age at birth and sex of child (data not shown). In addition, there was an increased incidence of leukaemia in parents of children with Down syndrome, OR=10.6 (95% CI 1.4–79.7). The OR was 2.0 (95% CI 1.0–4.1) for leukaemia in parents who had a child with any of the recorded malformations excluding Down syndrome (data not shown).

## DISCUSSION

We found no increased risk of cancer in parents after the birth of their first liveborn child with congenital malformations in general. We did, however, find a higher cancer risk in parents whose first liveborn child had cleft lip/palate, especially of lymphomas or leukaemia. Our prior hypotheses did not include specific malformations or specific cancers and this may be a chance finding due to multiple comparisons. The associations were on the other hand strong and consistent, in particular for maternal cancer. To the best of our knowledge, this is a novel observation and should encourage others to evaluate the associations we found. Most studies (Mulvihill *et al*, 1987; Dodds *et al*, 1993; Kenney *et al*, 1996; Byrne *et al*, 1998; Olsen and Storm, 1998) have found no association between cancer and malformations in two generations, but these studies have been too small to study specific malformations.

We used parents who had no cancer diagnosed until after their index children were born. Our study is thus not confounded by treatment for cancer, which might have teratogenic effects. We limited the analysis to firstborn children to avoid bias induced by the outcome of previous births, and to achieve statistical independence. We did not have data on stillbirths, which may bias our results if fetal mortality differs between parents who will develop cancer and parents who will not. Maternal age at birth and sex of child were taken into consideration in the analysis since some congenital malformations have been suggested to correlate with them (Hollier *et al*, 2000; Lary and Paulozzi, 2001).

The association between parental cancer and cleft lip/palate in offspring was persistent in time and not restricted to cancers diagnosed shortly after conception. It is, therefore, unlikely that the association is caused by early cancer lesions.

Genetic factors play a role in the cause of cleft lip/palate in addition to certain environmental factors, e.g. vitamins (Christensen and Fogh-Andersen, 1993; Shaw *et al*, 1995). Its inheritance is generally regarded as multigenic with allelic variation at different loci. Transforming growth factor-alpha (TGF alpha), transforming growth factor-beta 3 (TGF beta 3), and retinoic acid receptor alpha (RARA), have all been suggested as possible determinants (Chenevix-Trench *et al*, 1992; Christensen and Fogh-Andersen, 1993; Houdayer and Bahuau, 1998). Both TGF and RARA were found to interact with retinoic acid, a recognized teratogen for cleft palate (Raja *et al*, 1991). In addition, acute promyelocytic leukaemia has been associated with chromosomal translocations (15;17), which disrupts the RARA gene (Hauksdottir and Privalsky, 2001). TGF beta is a potent regulator of numerous processes including haematopoiesis, cell proliferation, differentiation and activation. TGF beta has pleiotropic and profound effects on the immune system and on haematologic malignancies, e.g. leukaemia (de Visser and Kast, 1999).

Cancer risks in children with malformations have been subject to research. The risk of leukaemia among children with Down syndrome is at least 10- to 20-fold higher than that among the general population (Fong and Brodeur, 1987; Mili *et al*, 1993; Hasle *et al*, 2000). The finding provides strong evidence for a gene or genes present on chromosome 21 with an important function in the development of leukaemia in individuals with Down syndrome (Kempski *et al*, 1997). Children with leukaemia have been found to be more likely than controls to have cleft lip/palate (Zack *et al*, 1991). In the present study, we found a significantly increased risk of leukaemia in parents of children with cleft lip/palate and in parents of children with Down syndrome.

Nonsyndromic cleft lip with or without cleft palate (NSCLP) is the most common type of cleft lip/palate with an average prevalence of 1.0 per 1000 live births. In a pilot study, it was found that cancer risks were 170–5000 times the expected occurrence in the first relatives of 49 NSCLP cases, 30–250 times the expected in their second relatives before the age of 50 years and 2–4 times the expected in their second relatives at the older ages (Yang *et al*, 1994). Another study found no significantly increased cancer risk in the first- or second-degree relatives of 196 NSCLP cases (Steinwachs *et al*, 2000). Our study provided better data for risk assessment.

The completeness of cleft lip and palate registration in the Hospital Discharge Registry has been found to be constant during the period 1983–1987 and to cover more than 90% of all known cases (Christensen *et al*, 1992; Christensen and Knudsen, 1998). Furthermore, the Danish cancer registry is believed to have almost complete ascertainment of cancers other than skin cancer (Storm *et al*, 1997).

Our results raise the possibility that parents of children with cleft lip/palate have an increased risk of developing lymphomas or leukaemia. It is notable that the risk estimates in general are highest among females. If further studies corroborate the finding this could lead to the formulation of new hypotheses concerning the aetiology of lymphomas, leukaemia and cleft lip/palate.

## References

[bib1] AndersenTFMadsenMJorgensenJMellemkjoerLOlsenJH1999 The Danish National Hospital Register–A valuable source of data for modern health sciences Dan Med Bull 46 263268 10421985

[bib2] ByrneJRasmussenSASteinhornSCConnellyRRMyersMHLynchCFFlanneryJAustinDFHolmesFFHolmesGEStrongLCMulvihillJJ1998 Genetic disease in offspring of long-term survivors of childhood and adolescent cancer Am J Hum Genet 62 4552 9443870 10.1086/301677 PMC1376803

[bib3] Chenevix-TrenchGJonesKGreenACDuffyDLMartinNG1992 Cleft lip with or without cleft palate: associations with transforming growth factor alpha and retinoic acid receptor loci Am J Hum Genet 51 13771385 1361101 PMC1682912

[bib4] ChristensenKHolmNVOlsenJKockKFogh-AndersenP1992 Selection bias in genetic-epidemiological studies of cleft lip and palate Am J Hum Genet 51 654659 1496993 PMC1682715

[bib5] ChristensenKFogh-AndersenP1993 Cleft lip (+/− cleft palate) in Danish twins, 1970–1990 Am J Med Genet 47 910916 8279491 10.1002/ajmg.1320470620

[bib6] ChristensenKKnudsenLB1998 Registration of congenital malformations in Denmark Dan Med Bull 45 9194 9504269

[bib7] ChristensenK1999 The 20th century Danish facial cleft population–epidemiological and genetic-epidemiological studies Cleft Palate Craniofac J 36 96104 10213053 10.1597/1545-1569_1999_036_0096_tcdfcp_2.3.co_2

[bib8] de VisserKEKastWM1999 Effects of TGF-beta on the immune system: implications for cancer immunotherapy Leukemia 13 11881199 10450746 10.1038/sj.leu.2401477

[bib9] DoddsLMarrettLDTomkinsDJGreenBShermanG1993 Case-control study of congenital anomalies in children of cancer patients BMJ 307 164168 8343744 10.1136/bmj.307.6897.164 PMC1678343

[bib10] FongCTBrodeurGM1987 Down's syndrome and leukemia: epidemiology, genetics, cytogenetics and mechanisms of leukemogenesis Cancer Genet Cytogenet 28 5576 2955886 10.1016/0165-4608(87)90354-2

[bib11] HasleHClemmensenIHMikkelsenM2000 Risks of leukemia and solid tumours in individuals with Down's syndrome Lancet 355 165169 10675114 10.1016/S0140-6736(99)05264-2

[bib12] HauksdottirHPrivalskyML2001 DNA recognition by the aberrant retinoic acid receptors implicated in human acute promyelocytic leukemia Cell Growth Differ 12 8598 11243468 PMC2712924

[bib13] HollierLMLevenoKJKellyMAMCIntireDDCunninghamFG2000 Maternal age and malformations in singleton births Obstet Gynecol 96(5 Pt 1)701706 11042304 10.1016/s0029-7844(00)01019-x

[bib14] HoudayerCBahuauM1998 Orofacial cleft defects: inference from nature and nurture Ann Genet 41 89117 9706339

[bib15] InfantePFWagonerJKWaxweilerRJ1976 Carcinogenic, mutagenic and teratogenic risks associated with vinyl chloride Mutat Res 41(1 spel. no)131141 1012297 10.1016/0027-5107(76)90083-x

[bib16] KempskiHMChessellsJMReevesBR1997 Deletions of chromosome 21 restricted to the leukemic cells of children with Down syndrome and leukemia Leukemia 11 19731977 9369434 10.1038/sj.leu.2400826

[bib17] KenneyLBNicholsonHSBrasseuxCMillsJLRobisonLLZeltzerLKMeadowsATReamanGHByrneJ1996 Birth defects in offspring of adult survivors of childhood acute lymphoblastic leukemia. A Childrens Cancer Group/National Institutes of Health Report Cancer 78 169176 8646713 10.1002/(SICI)1097-0142(19960701)78:1<169::AID-CNCR23>3.0.CO;2-X

[bib18] LaryJMPaulozziLJ2001 Sex differences in the prevalence of human birth defects: a population-based study Teratology 64 237251 11745830 10.1002/tera.1070

[bib19] McLachlanJANewboldRRBurowMELiSF2001 From malformations to molecular mechanisms in the male: three decades of research on endocrine disrupters APMIS 109 263272 11469497 10.1034/j.1600-0463.2001.d01-119.x

[bib20] MiliFKhouryMJFlandersWDGreenbergRS1993 Risk of childhood cancer for infants with birth defects. I. A record-linkage study, Atlanta, Georgia, 1968–1988 Am J Epidemiol 137 629638 8470664 10.1093/oxfordjournals.aje.a116720

[bib21] MulvihillJJMcKeenEARosnerFZarrabiMH1987 Pregnancy outcome in cancer patients. Experience in a large cooperative group Cancer 60 11431150 3607730 10.1002/1097-0142(19870901)60:5<1143::aid-cncr2820600537>3.0.co;2-e

[bib22] OlsenJStormH1998 Pregnancy experience in women who later developed estrogen-related cancers (Denmark) Cancer Causes Control 9 653657 10189052 10.1023/a:1008831802805

[bib23] RajaRHPatersonAJShinTHKudlowJE1991 Transcriptional regulation of the human transforming growth factor-alpha gene Mol Endocrinol 5 514520 1922084 10.1210/mend-5-4-514

[bib24] ShawGMLammerEJWassermanCRO'MalleyCDTolarovaMM1995 Risks of orofacial clefts in children born to women using multivitamins containing folic acid periconceptionally Lancet 346 393396 7623568 10.1016/s0140-6736(95)92778-6

[bib25] SteinwachsEFAmosCJohnstonDMullikenJStalSHechtJT2000 Nonsyndromic cleft lip and palate is not associated with cancer or other birth defects Am J Med Genet 90 1724 10602112 10.1002/(sici)1096-8628(20000103)90:1<17::aid-ajmg4>3.0.co;2-9

[bib26] StormHHMichelsenEVClemmensenIHPihlJ1997 The Danish Cancer Registry—history, Content, quality and use Dan Med Bull 44 535539 9408738

[bib27] The Danish National Board of Health, Statistical Office1999 Cancer incidence in Denmark 1996Copenhagen: Stougard Jensen

[bib28] Titus-ErnstoffLHatchEEHooverRNPalmerJGreenbergERRickerWKaufmanRNollerKHerbstALColtonTHartgeP2001 Long-term cancer risk in women given diethylstilbestrol (DES) during pregnancy Br J Cancer 84 126133 11139327 10.1054/bjoc.2000.1521 PMC2363605

[bib29] VainioH1989 Carcinogenesis and teratogenesis may have common mechanisms Scand J Work Environ Health 15 1317 2537999 10.5271/sjweh.1889

[bib30] VorobtsovaIE1989 Increased cancer risk as a genetic effect of ionizing radiation IARC Sci Publ 96 389401 2680955

[bib31] YangPMitchellESchwartzAGMulvihillJJHoggeA1994 Do cancers aggregate in families ascertained through probands with cleft lip and/or palate? Am J Hum Genet 55suppl A97

[bib32] ZackMAdamiHOEricsonA1991 Maternal and perinatal risk factors for childhood leukemia Cancer Res 51 36963701 2065325

